# Clinical Significance of Peritoneal Fluid Examination for Free Cancer Cells in Patients Qualified for Surgery for Gastric Cancer

**DOI:** 10.3389/fsurg.2021.685868

**Published:** 2021-06-21

**Authors:** Sławomir Cieśla, Radosław Lisiecki, Agnieszka Ławnicka, Bartosz Kudliński, Paulina Ostrowska, Alberto Davì, Massimiliano Veroux, Dawid Murawa

**Affiliations:** ^1^General and Oncological Surgery Clinic of the K. Marcinkowski University Hospital in Zielona Góra, Zielona Góra, Poland; ^2^Department of General and Oncological Surgery of the Medical Centre in Pleszew, Pleszew, Poland; ^3^Department of Anaesthesiology, Intensive Care and Emergency Medicine at Collegium Medicum of the University of Zielona Góra, Zielona Góra, Poland; ^4^Department of Oncology of the Medical Centre in Pleszew, Pleszew, Poland; ^5^Vascular and Endovascular Surgery Unit, Cuneo, Italy; ^6^General Surgery Unit, Department of Medical and Surgical Sciences and Advanced Technologies University Hospital of Catania, Catania, Italy; ^7^Department of Surgery and Oncology, Faculty of Medicine and Health Sciences of the University of Zielona Góra, Zielona Góra, Poland

**Keywords:** gastric cancer, resection, gastrectomy, sub-total gastrectomy, free cancer cell, peritoneal lavage, survival, prognosis

## Abstract

**Background:** Peritoneal lavage cytology in patients with gastric cancer may correlate with an unfavorable prognosis. This study evaluated the presence of free cancer cells in the peritoneal lavage of a population of patients with gastric cancer and its correlation with the outcome of surgical treatment.

**Methods:** One hundred patients diagnosed with gastric or gastrointestinal junction adenocarcinoma underwent surgery. In all patients, a cytological and immunohistochemical analysis of peritoneal lavage was performed. Based on the presence of free cancer cells (fcc) at the cytological and immunohistochemical examination of peritoneal lavage, patients undergoing surgery for gastric cancer were divided into two groups: fcc (+) and fcc (–).

**Results:** A total of 100 patients, 37 women, and 63 men with a median age of 65 years were included in the study. In the entire study group, 16 (16%) patients were positive for the presence of free cancer cells (fcc +) at peritoneal lavage examination. However, in the group of patients who underwent gastrectomy, fcc (+) was found in 10 out of 77 (13%) patients. The presence of cancer cells in peritoneal lavage was a strong predictive factor in an unfavorable outcome after surgery, and 1-year and 2-year patient survival was 34 and 0% in fcc (+) patients and 79 and 59% in fcc (–), respectively. Moreover, the presence of free cancer cells was associated with a five-fold increased risk of death within 2 years after surgery. When analyzing the group of patients undergoing R0/R1 surgery, this difference was even more significant (*p* < 0.0001).

**Conclusions:** The presence of free cancer cells in peritoneal lavage may significantly affect the outcome of patients with gastric cancer. Radical surgery in patients with free cancer cells in the peritoneal lavage did not result in a survival advantage. Identification of free cancer cells could help for a better stratification of gastric cancer patients, identifying those patients who could better benefit from a radical surgical treatment, finally improving long-term survival.

## Introduction

Gastric cancer carries a poor prognosis. More than half of patients undergoing radical surgery for poorly differentiated T3/T4 gastric cancer experience cancer recurrence in the form of peritoneal dissemination (1–3). The most important risk factors in peritoneal dissemination are an advanced stage of the disease at diagnosis, an unfavorable histological subtype, an R1 surgical resection and the presence of free cancer cells in the peritoneal lavage fluid (1–3). In 2010, the Union for International Cancer Control (UICC) included the result of peritoneal lavage cytology in the seventh edition of the TNM classification (4). Patients with positive cytology are classified as an M1 category and, at the same time, as stage IV cancer (5).

Many basic methods of peritoneal lavage analysis have been described in the literature: The classical cytology involving examination of a smear made from a cell pellet on the microscope slide and a pigment assessment conducted by an experienced pathologist (6), the immunohistochemical method using the reaction of antibodies against antigens present on cancer cells (7), the immunoenzymatic method assessing the CEA level in the supernatant after centrifugation of lavage fluid (8), and molecular methods involving the identification of cancer cell genes employing RT-PCR techniques (9). These methods differ in sensitivity and specificity in predicting peritoneal recurrence, and cytology remains the gold standard for peritoneal lavage examination (10) due to its simplicity in implementation, the short analysis time, low costs, and its high specificity in predicting peritoneal recurrence. Unfortunately, cytology has a low sensitivity, with many patients with negative peritoneal cytology experiencing a peritoneal cancer recurrence after radical surgery (11). The European Society for Medical Oncology (ESMO) recommends the peritoneal lavage analysis in all patients with a potentially resectable gastric cancer (stages IB–III) (12).

The aim of the study is to evaluate the presence of free cancer cells in the peritoneal lavage of patients with gastric cancer, with the aim of correlating the result of peritoneal lavage examination with the outcome of surgical treatment of gastric cancer.

## Materials and Methods

### Study Population

In the period from 1.07.2014 to 31.12.2016, 100 patients diagnosed with gastric or gastrointestinal junction adenocarcinoma underwent surgery. In all patients, a cytological and immunohistochemical analysis of peritoneal lavage was performed.

In all patients, complete cancer stadiation was obtained before surgery: All the patients underwent upper gastrointestinal tract endoscopy, abdominal CT, x-ray, or CT of the chest. In the preoperative assessment, endosonography and diagnostic laparoscopy were not performed as standard. In patients with advanced stage cancer, the preoperative assessment was completed with PET/CT scan. Peritoneal lavage examination was performed, regardless of the type of surgery. After opening the peritoneal cavity, 300 ml of saline at 37°C was administered into the area of the stomach cancer. After 30 s, 100 ml of lavage fluid was recovered and immediately transferred to the Department of Pathology for cytological and immunohistochemical examination. In the first stage, the liquid underwent centrifugation in order to separate the pellet. Cytological preparations obtained from the pellet were then stained, using hematoxylin and eosin dyes. The remaining portion of the pellet was used to prepare paraffin cytoblocks. Next, 4.5-μm-thick paraffin sections were cut from the obtained cytoblocks, and they were used for further basic and immunohistochemical staining, using primary antibodies against Ber-EP4, CK7/20, and B72.3, and the EnVisio (Dako) detection system.

Surgical techniques utilized were the following ones:

- **D2 total gastrectomy**- The whole stomach is removed. During this type of operation, lymphadenectomy is also performed. Lymph nodes dissected in D1 include nodes in stations 1 to 7; D1 positive (D1+) includes nodes in D1 stations and 8a, 9, and 11 p; D2 includes nodes in D1 stations and 8a, 9, 10, 11p, 11d, and 12a. Station 10 lymph node dissection may be omitted (13).
- **D2 extended total gastrectomy**- This operation is indicated in the treatment of gastric cancer for necessity or to achieve an oncologic radicality. By this surgical treatment, the stomach and other organs or a part of them involved by a primitive tumor are removed (14).
- **Subtotal resection**- is the treatment of choice for middle and distal-third gastric cancer as it provides similar survival rates and a better functional outcome compared with total gastrectomy, especially in early-stage disease with a favorable prognosis (15).
- **Laparotomy/gastric bypass**- is a surgical procedure that creates an anastomosis between the stomach and the jejunum (16).

Radicality of surgical treatment was assessed with the R classification (16). The R classification denotes the absence or presence of a residual tumor after treatment. R0 corresponds to resection for cure or complete remission. R1 to a microscopic residual tumor, R2 to a macroscopic residual tumor (17, 18).

### Statistical Analysis

Based on the presence of free cancer cells (fcc) at the cytological and immunohistochemical examination of peritoneal lavage, patients undergoing surgery for gastric cancer were divided into two groups: fcc (+) and fcc (–). The results of the peritoneal analysis were compared with the information obtained from imaging, endoscopic, and histopathological examinations. Subsequently, survival times in the fcc (+) and the fcc (–) groups were compared. A survival analysis was performed within the analyzed groups, employing the log-rank test. The obtained results were presented as the Kaplan–Meier curves. A logistic regression model was applied for prognostic assessment of individual factors, determining the occurrence of fcc (+) in all patients. The results were presented as an odds ratio with a 95% confidence interval. All tests were analyzed at the significance level of α = 0.05. Calculations were performed, using the Statistica 10 statistical package from StatSoft and MedCalc version 10.3.2 (MedCalc Software, Mariakerke, Belgium).

## Results

A total of 100 patients undergoing surgery for gastric cancer were included in this study. The study group included 37 women and 63 men, with a median age of 65 years (range 26–81 years) (Table 1). In the entire study group, 16 (16%) patients were positive for the presence of free cancer cells (fcc +) at peritoneal lavage examination. However, in the group of patients who underwent gastrectomy, fcc (+) was found in 10 out of 77 (13%) patients. In 23 patients, the peritoneal spread of cancer or local advancement was observed during laparotomy, which prevented gastrectomy. In this group, a positive result of peritoneal lavage examination was obtained in six patients (26%).

**Table 1:** Clinical and histopathological characteristics of the study group.

	***N* = 100 (%)**	
**Age (mean, ys)**	63.8	
**Female/Male**	37 (37)/63(63)	
**Type of surgery (*****N*****, %)**		
	D2 total gastrectomy	69 (69)
	D2 extended total gastrectomy	4 (4)
	Subtotal resection	4 (4)
	Laparotomy/gastric bypass	23 (23)
**Radicality of resection (*****N*****, %)**		
	R0 resection	68 (68)
	R1 resection	8 (8)
	R2 resection	1(1)
**Cancer location (*****N*****, %)**		
	Proximal part of stomach (the cardia and/or bottom of stomach)	22 (22)
	Distal part (body and/or prepyloric region)	64(64)
	Entire stomach	14(14)
**Degree of histological malignancy (*****N*****, %)**		
	G1	5 (5)
	G2	32 (32)
	G3	63(63)
**Type of Gastric Cancer according to Lauren Classification (17)**		
	Intestinal	43 (43)
	Diffuse	44 (44)
	Mixed	13(13)
**Stage of cancer (TNM)** **(****4****)**	I	15(15)
	IIA	16 (16)
	IIB	10 (10)
	IIIA	14 (14)
	IIIB	18 (18)
	IIIC	6 (6)
	IV	21(21)
T parameter		
	T1	10 (10)
	T2	13 (13)
	T3	43 (43)
	T4a	26(26)
	T4b	8 (8)
N parameter		
	N+	66 (66)
	N-	34 (34)
**Macroscopically visible peritoneal/organ dissemination**	M1	21 (21)
**Perioperative/postoperative treatment**	Perioperative chemotherapy	36 (36)
	Postoperative chemotherapy	22 (2)
	Palliative chemotherapy	18 (18)

Cytological examination of peritoneal lavage was performed in all patients, while immunohistochemistry was performed only in 46 patients (Table 2).

**Table 2:** Results of peritoneal lavage examination for the presence of free cancer cells according to the diagnostic methods.

**Diagnostic method**	**Number of patients**	**fcc (–)**	**fcc (+)**	**%**
Cytology	100	86/100	14/100	14
Immunohistochemistry	46	34/46	8/46	17
Cytology and/or immunohistochemistry	100	84/100	16/100	16

Among the 16 fcc (+), 8 (50%) were positive at cytology, 2 (12%) at immunochemistry and 6 (38%) at both cytology and immunochemistry. The mean survival of the fcc (+) group was 9 months, while, in fcc (–), the mean survival of patients was 22 months, with 59% of patients surviving 2 years after surgery. Patients with fcc (+) had a five-fold higher risk of death [Odds Ratio (OR), 5.16, a 95% confidence interval (CI), 1.9–14.1]; Moreover, fcc (+) patients undergoing gastric cancer surgery had significantly lower survival compared with fcc (–) patients: 1-year and 2-year patient survival was 34 and 0% in fcc (+) patients and 79 and 59% in fcc (–), respectively.

**Figure 1:**
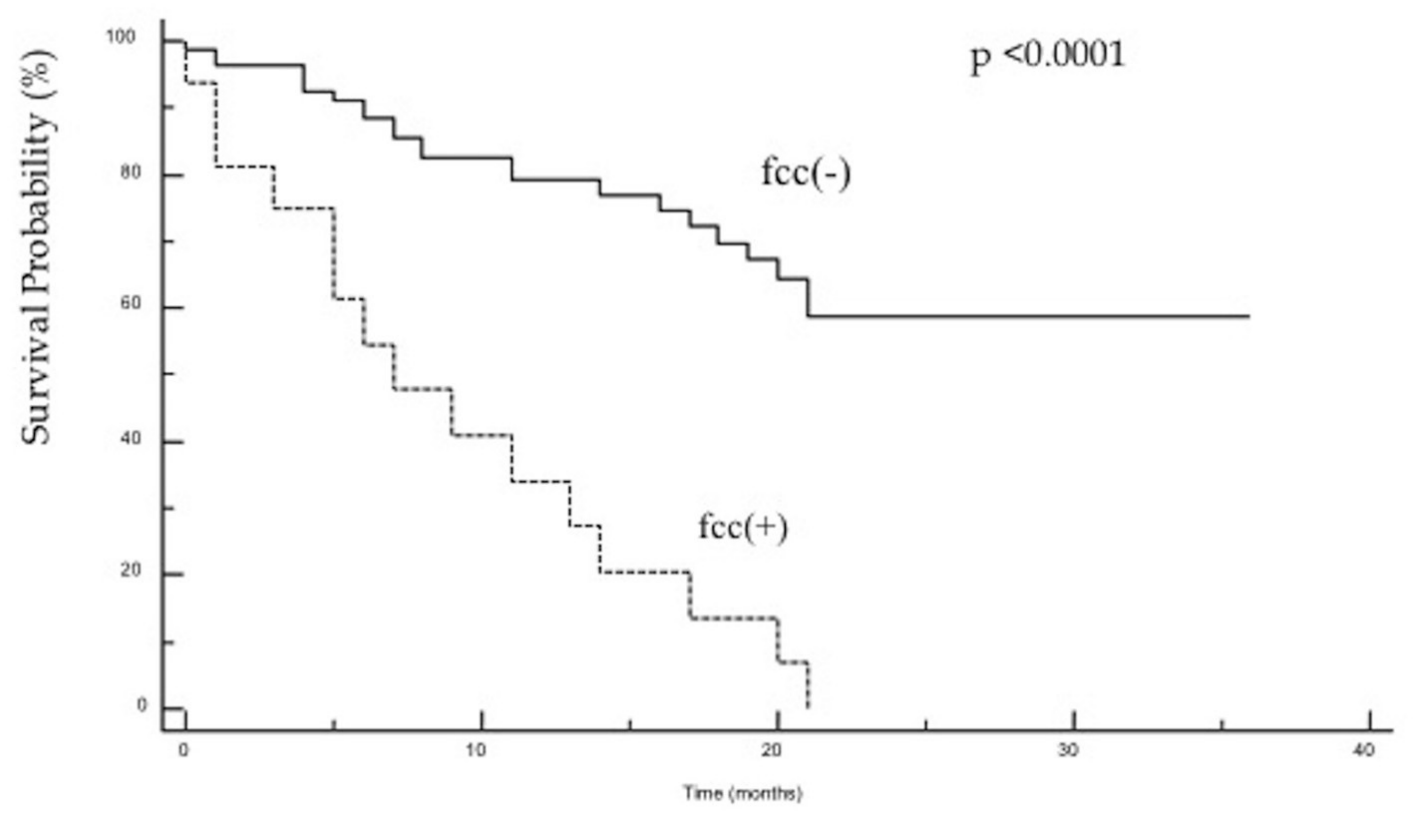
A Kaplan–Meier survival curve for the entire study group, comparing patients with fcc (+) [hazard ratios (HR) 5.161 (95% CI: 1.884–14.135)] and fcc (–) [HR.193 (95% CI: 0.070–0.530), *p* < 0.0001].

**Figure 2:**
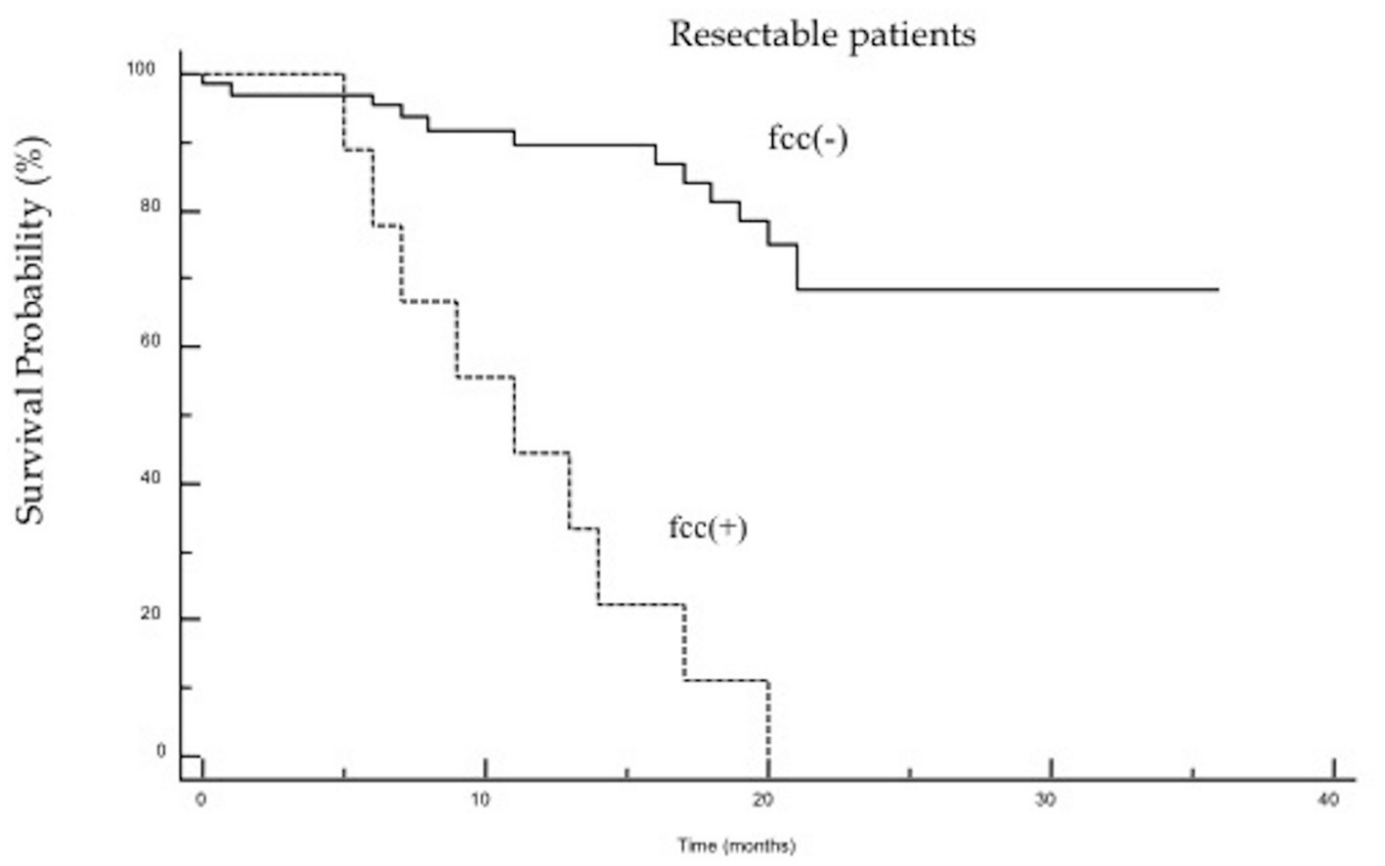
The Kaplan–Meier survival curves for resected patients, comparing patients with fcc (+) [HR7.799 (95% CI: 1.693–35.915)] and fcc (–) [HR.128 (95% CI: 0.027–0.590), *p* < 0.0001].

**Figure 3:**
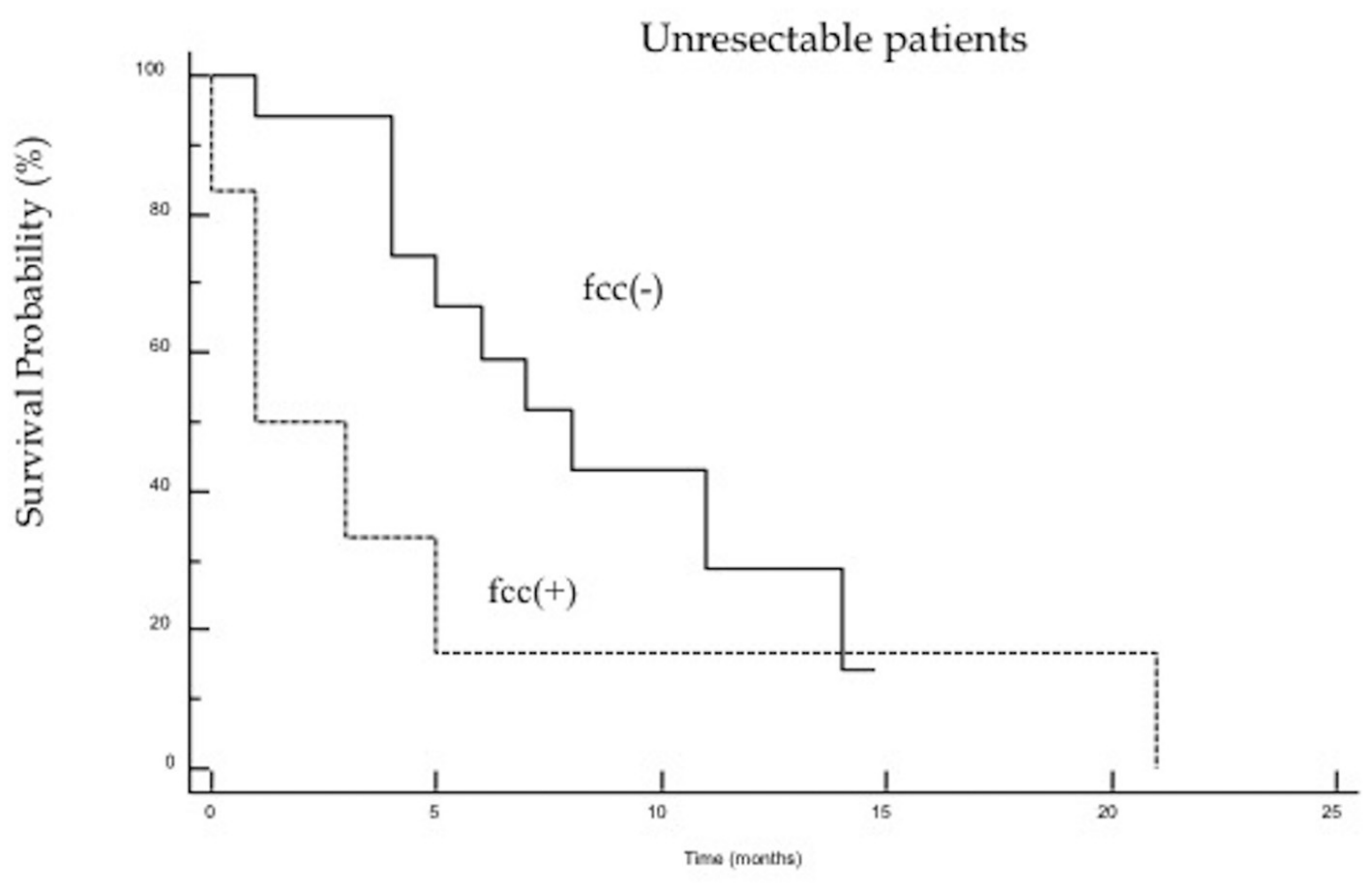
The Kaplan–Meier survival curves for non-resected patients from fcc (+) [HR 1.962 (95% CI: 0.617–6.242)] and fcc (–) groups [HR.509 (95% CI: 0.160–1.620), *p* = 0.11].

**Figure 4:**
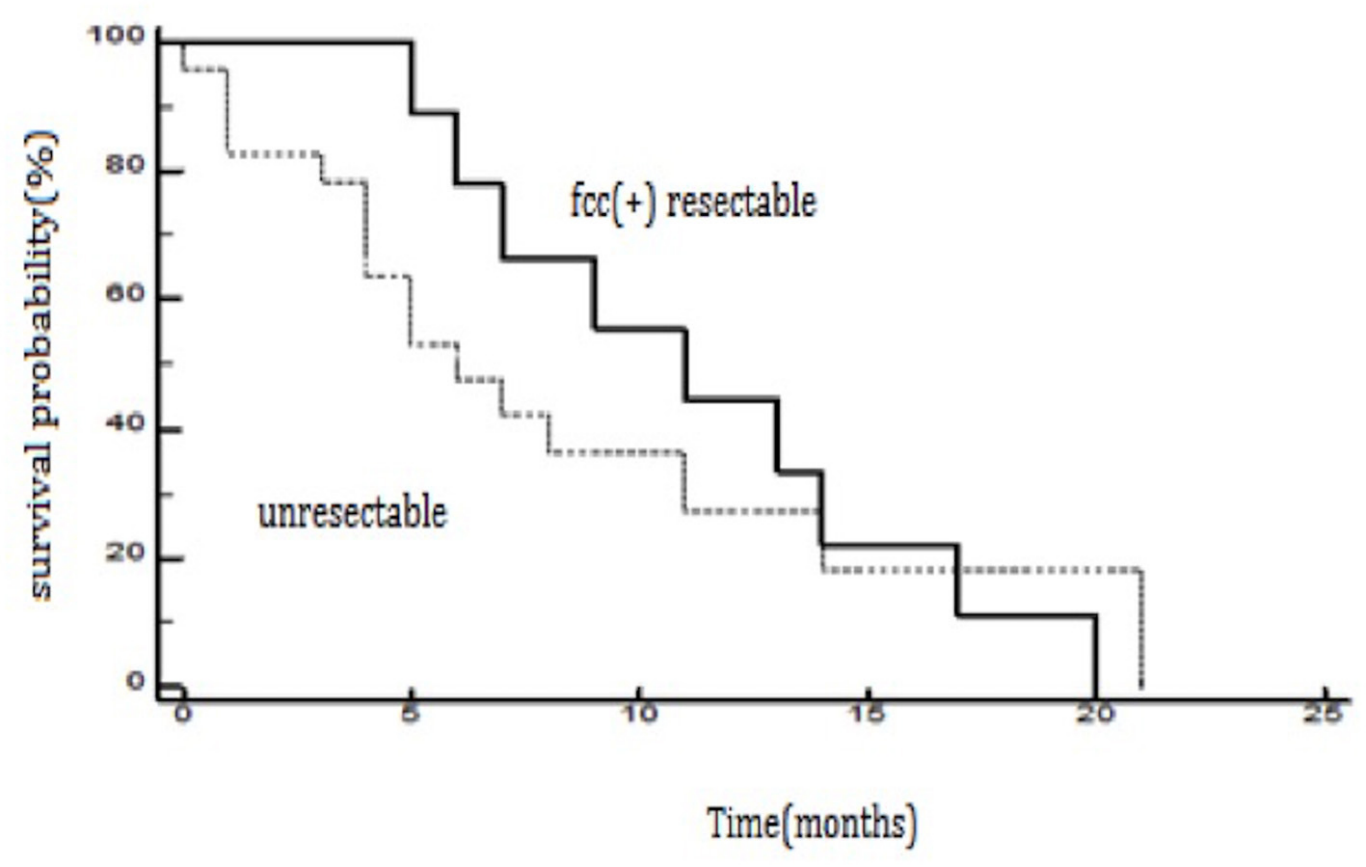
The Kaplan–Meier survival curves for resected patients from the fcc (+) group and non-resected patients from the fcc (+) and fcc (–) group (*p* = 0.47).

The study demonstrated that the presence of cancer cells in peritoneal lavage is a strong predictive factor in an unfavorable outcome after surgery and is associated with a five-fold increased risk of death in the first 2 years after surgery (Figure 1). When analyzing the group of patients undergoing R0/R1 surgery, this difference was even more significant (*p* < 0.0001), and the risk of death in the first 2 years in the fcc (+) group was significantly higher (OR 7.8; 95% CI 1.7–35.9) than in the fcc (–) group (Figure 2). In this subgroup of patients, 1-year and 2-year patient survival was 44 and 0% for fcc (+) and 89 and 68% for fcc (–), respectively. In the subgroup of non-resected patients, even considering the small number of included patients, the difference in survival between fcc (+) and fcc (–) was not statistically significant (*p* = 0.11) (Figure 3), suggesting that it should not yet be possible to consider whether FCC+/– is relevant in unresectable patients. When evaluating the survival of patients with free cancer cells in the peritoneal lavage undergoing surgery, this study demonstrated that there was no significant difference in survival among fcc (+) patients and patients unsuitable for surgical resection, irrespective of the presence of free cancer cells in the peritoneal lavage (*p* = 0.47). The mean survival time in the group of non-resected patients was 9 months, whereas, in the resected fcc (+) group, it was 11 months (Figure 4). The multifactorial analysis and the logistic regression model demonstrated that free cancer cells in the peritoneal lavage are more frequently associated with poorly differentiated G3 cancer (OR 5, 95% CI 1.068–23.412, *p* = 0.04), with diffuse-type according to Lauren's criteria (OR 7.04, 95% CI 1.862–26.636, *p* = 0.004), and when the cancer process affected the entire stomach (*p* < 0.001). On the other hand, cancer to the distal part of the stomach and a histological intestinal type, according to Lauren's classification, is not associated with an increased risk of positive peritoneal lavage examination. Paradoxically, the presence of peritoneal metastases at the time of surgery is not correlated with an increased incidence of free cancer cells on peritoneal lavage (*p* = 0.27) (Table 3).

**Table 3:** Logistic regression of parameters, which may affect the positive result of peritoneal lavage examination.

**Parameter**		**Odds ratio (HR)**	**Confidence interval −95%+95%**	**Standard error**	***p***
Location	Cardia	0.808	0.207–3.148	0.634	0.758
	Body and/or prepyloric region	0.120	0.035–0.412	0.629	<0.001
	Entire stomach	19.800	5.188–75.568	0.683	<0.001
Degree of differentiation	G1	Assessment not possible – G1 was not present in the fcc (+) group			
	G2	0.257	0.055–1.208	0.789	0.085
	G3	5.000	1.068–23.412	0.788	0.041
Type according to Lauren	Intestinal	0.150	0.032–0.700	0.787	0.015
	Diffuse	7.042	1.862–26.636	0.679	0.004
	Mixed	0.442	0.052–3.690	1.082	0.451
T3/T4		Not determined—all fcc (+) patients had T3/T4 stage			
N+		Not determined—all patients from fcc (+) group had N (+) stage			
Macroscopically visible peritoneal dissemination		1.932	0.588–6.345	0.607	0.277

## Discussion

The presence of free cancer cells in peritoneal lavage has been reported in the literature with a frequency ranging from 4.4 to 83% (19), depending on the analyzed group of patients (R0 resection vs. peritoneal dissemination) and on the applied methods of cell identification.

This study reported an incidence of 13% positive peritoneal cytology among patients undergoing R0 resection for gastric cancer, similar to that reported in the literature, where a positive peritoneal lavage has been reported with an incidence ranging from 4.4 to 11% (20–23).

This difference may be partially explained also to the different methods of analysis of peritoneal lavage: Benevolo et al. (24) reported a 14% increased rate of free cancer cells detection with the immunohistochemical method compared with cytology, and, in the group of patients identified solely by the immunohistochemical method, they observed similar rates of recurrence and distant survival compared with the group of patients with a positive cytological examination (24). These results were confirmed by other studies, which reported an incidence of fcc ranging from 21.4 to 30% (25–27).

In our study, we did not observe such an obvious benefit of using the immunohistochemical method. When used alone, the immunochemistry method confirmed the presence of fcc in only 2 of 10 patients undergoing R0/R1 resection, probably as the consequence of the lack of sufficient cell material to perform the examination in all patients using the two methods simultaneously, suggesting that the results of immunohistochemistry should be interpreted with caution.

The presence of free cancer cells in peritoneal lavage usually precedes peritoneal dissemination. Therefore, the low-detection rate (23.8%) obtained in the group of patients with coexisting macroscopically visible peritoneal dissemination is puzzling. Similar findings were reported in the literature with conflicting incidence (23–83%), probably as a consequence of the sensitivity of the diagnostic methods and the lack of standardization of lavage examination (20, 28–30).

This study clearly demonstrated that fcc (+) patients undergoing gastric cancer surgery had significantly lower survival compared with fcc (–) patients: 1-year and 2-year patient survival was 34 and 0% in fcc (+) patients and 79 and 59% in fcc (–), respectively. In the subgroup of patients who underwent a R0 resection, 1-year and 2-year patient survival was 44 and 0% for fcc (+) and 89 and 68% for fcc (–), respectively. Bando et al. (20) in their study analyzed cytology of peritoneal fluid in 1,297 patients who underwent surgery for gastric cancer. Cytological examination of peritoneal lavage was positive in 296 patients (24%); among positive patients, only 2% of patients survived 5 years after surgery, compared with the 58% of patients with negative cytology (*p* < 0.001). Patients with positive cytology were further divided into two groups; in patients undergoing a potentially curative gastric resection, 1-year and 3-year patient survival was 37 and 0%, respectively, while, in patients with peritoneal dissemination, 1-year and 3-year survival was 18 and 2%, respectively (20). In contrast, 1-year and 3-year survival in patients with negative cytology was 43 and 9%, respectively (*p* < 0.001) (20). Fukugawa et al. (31) observed a similarly adverse effect on survival in patients with a positive result of peritoneal lavage examination and concomitant dissemination of the disease.

Notably, our analysis was not able to confirm such findings: in patients with advanced disease, 1-year and 2-year survival (17 and 0%, respectively) of fcc (+) was not significantly different from that of fcc (–) patients (29 and 0%, respectively).

There are conflicting survival rates reported in the literature for patients with fcc in peritoneal lavage. While some authors (28, 32, 33) report no patient survival at 2-year follow-up, other authors reported 2-year survival, ranging from 60 to 75% (25, 34, 35). Again, the differences in patient survival could be attributed to the different examination methodology and in the evaluation of cytological findings.

The peritoneal dissemination, defined as only the presence of cancer cells in peritoneal lavage without macroscopically visible peritoneal dissemination, indicates an advanced disease poorly sensible to surgical treatment. In their study, Nath et al. (30) evaluated the survival of patients with peritoneal dissemination, evaluated during a diagnostic laparoscopy. Patients with macroscopically visible dissemination, regardless of the result of lavage examination, and patients who had a positive result but no visible metastases in the peritoneum were not considered suitable for surgical treatment. The survival analysis did not demonstrate statistically significant differences in survival of patients with peritoneal dissemination in relation to patients with positive peritoneal cytology only (9 vs. 13 months; *p* = 0.517) (29). Some authors suggested that laparoscopic exploration with peritoneal lavage could be useful for patients with advanced disease (T3–T4), with G3 differentiation of diffuse-type according to Lauren classification (36). However, the Japanese Gastric Cancer Association guidelines (5th edition) suggest that staging laparoscopy in patients at high risk of peritoneal dissemination has limited indications in the decision-making for neoadjuvant chemotherapy (37).

Patients in stage IV cancer are not usually scheduled for surgical treatment, since there is no significative survival advantage of surgery with adjuvant chemotherapy compared with chemotherapy alone (38, 39).

Many studies suggested a potential beneficial effect of gastrectomy in fcc (+) patients without peritoneal dissemination. Miyashiro et al. (40) evaluated the potential survival benefit of gastrectomy in patients with fcc (+) compared with patients with peritoneal dissemination who did not undergo resection. The average lifetime in these groups was 18.4 and 10.8 months, respectively (*p* = 0.018). The 3-year survival of fcc (+) patients who underwent gastrectomy was 24%, whereas, in the case of peritoneal dissemination, this rate was 4% (40). Fukugawa (31) presented similar benefits from gastric resection in fcc (+) patients. He achieved a 2- and 5-year survival rate of 25.3 and 7.8%, respectively, in the fcc (+) group, significantly better (*p* = 0.002) compared with non-resected patients with peritoneal dissemination. In addition, the authors did not report benefits from adjuvant chemotherapy (based on 5-fluorouracil) in the fcc (+) resected group (*p* = 0.123) (31), and a chemotherapy-driven change of peritoneal lavage from fcc (+) to fcc (–) has been associated with an improvement in a prognosis (19, 41, 42). Our analysis showed that the mean survival of fcc (+) patients undergoing a radical resection was 11 months, compared with the 8.5-month average survival time of non-resected patients, regardless of the lavage result (*p* = 0.47). It should be noted that, in 8 out of 10 patients treated with neoadjuvant chemotherapy, the peritoneal lavage during surgery was positive, suggesting a lack of response to the treatment, and this highlights the need for accurate preoperative evaluation of peritoneal lavage for correct planning of the surgical procedure.

In our study, we evaluated the factors that could increase the likelihood of positive peritoneal lavage. All the patients with fcc (+) at peritoneal lavage had advanced stage gastric cancer; in the patients with ≥ T3 stage, 21% of the patients had positive cytology, while, in the patients with lymph nodes metastases, this incidence raised to 24%.

The correct identification of tumor cells in the free peritoneal cavity of gastric cancer patients may be useful to identify those patients potentially suitable for total gastrectomy after S-1 monotherapy (oral fluoropyrimidine) (43) or for hyperthermic intraperitoneal chemotherapy (HIPEC) (37). HIPEC may reduce intraperitoneal cancer recurrence, potentially improving patient survival (44–47).

The Dutch Gastric Cancer Trial (22) demonstrated that the tumor invasion to the serosa was associated with an 11-fold higher risk of positive peritoneal cytology, and the involvement of the lymphatic system was associated with a five-fold increased risk of positive cytology. Similar results were published by La Torre et al. (48), who demonstrated that, among those with fcc in the peritoneal lavage, 86% of the patients were diagnosed with T3/T4 cancer, and all had lymph node metastases. The analysis of the study group demonstrated that, in the patients with T3 grade, peritoneal lavage examination was positive in 25% of the patients, whereas, when lymph node metastases were present, this rate amounted to 19.4% (48), confirming that both the advanced stage of the tumor and the presence of lymph node metastases increase the risk of finding fcc in the peritoneal lavage.

On the other hand, some authors (19, 20) found a relationship between the lavage result and the location of cancer in the stomach: The “linitis plastica” type of cancer and its location in the gastroesophageal junction were more significantly associated with a positive result of lavage examination, as reported in our study, where the linitis plastica was associated with >20-fold higher risk of positive cytology, while we did not observe significantly more frequent positive results for the location of cancer in the proximal part of the stomach. There are also studies demonstrating the dependence of the lavage examination result on the size of the tumor (49). Suzuki et al. presented data showing that, in the fcc (+) group, the tumor diameter was, on average, 9.7 cm compared with 4.7 cm in fcc (–) patients (50).

We also found that the diffuse type of gastric cancer, according to the Lauren's classification, is associated with an increased risk of positive peritoneal lavage. These results are in contrast with these reported by other authors (37, 41, 49), who did not confirm a positive correlation between the diffuse type and the positive result of lavage examination.

Although this study reported many important findings, we are aware of its potential limits; although some authors reported similar findings, this study highlights that the prognosis of fcc (+) patients are extremely poor, even after radical surgery. This study is retrospective in nature, but it entails a single-center experience, thereby eliminating potential confounding factors, such as different surgical procedures and adjuvant therapies. Moreover, this study provides many important findings that could be useful for developing future prospective studies.

## Conclusion

The presence of free cancer cells in peritoneal lavage is a strong negative prognostic factor in patients with gastric cancer. Radical surgery in patients with free cancer cells in the peritoneal lavage did not result in a survival advantage, suggesting that the peritoneal lavage examination should be included in the preoperative evaluation for correct planning of the surgical treatment. The presence of free cancer cells is strongly related to the stage and the type of gastric cancer. Identification of free cancer cells could help for a better stratification of gastric cancer patients, identifying those patients who could better benefit from a radical surgical treatment, finally improving long-term survival.

## Data Availability Statement

The raw data supporting the conclusions of this article will be made available by the authors, without undue reservation.

## Ethics Statement

The studies involving human participants were reviewed and approved by Marcinkowski University Bioethics Committee at the Poznan University of Medical Sciences. The patients/participants provided their written informed consent to participate in this study.

## Author Contributions

SC, RL, AŁ, and DM: substantial contributions to the conception and design of the work. SC, RL, AŁ, PO, and AD: acquisition, analysis, and interpretation of the data for the work. MV and DM: drafting of the work and revising it critically for important intellectual content. All authors approved the final version of the manuscript.

## Conflict of Interest

The authors declare that the research was conducted in the absence of any commercial or financial relationships that could be construed as a potential conflict of interest.

## Abbreviations

FCC: free cancer cells
UICC: Union for International Cancer Control
CT: Computed tomography.
